# Maternal elevated inflammation impairs placental fatty acids β-oxidation in women with gestational diabetes mellitus

**DOI:** 10.3389/fendo.2023.1146574

**Published:** 2023-05-05

**Authors:** Francisco Visiedo, Luis Vázquez-Fonseca, Jessica Ábalos-Martínez, J. Román Broullón-Molanes, Rocío Quintero-Prado, Rosa María Mateos, Fernando Bugatto

**Affiliations:** ^1^ Inflammation and Metabolic Syndrome in Pregnancy Group (CO25), Biomedical Research and Innovation Institute of Cádiz (INiBICA), Cádiz, Spain; ^2^ Division of Maternal and Fetal Medicine, Department of Obstetrics and Gynecology “Puerta del Mar” University Hospital, University of Cádiz, Cádiz, Spain; ^3^ Area of Obstetrics and Gynaecology, Department of Child and Mother Health and Radiology, School of Medicine, University of Cádiz, Cádiz, Spain; ^4^ Department of Obstetrics and Gynecology, Puerto Real University Hospital, Cadiz, Spain; ^5^ Area of Biochemistry and Molecular Biology, Department of Biomedicine, Biotechnology and Public Health, University of Cádiz, Cádiz, Spain

**Keywords:** gestational diabetes mellitus, fatty acid oxidation, proinflammatory milieu, lipid metabolism, pregnancy, placenta

## Abstract

**Introduction:**

An adverse proinflammatory milieu contributes to abnormal cellular energy metabolism response. Gestational diabetes mellitus (GDM) is closely related to an altered maternal inflammatory status. However, its role on lipid metabolism regulation in human placenta has not yet been assessed. The aim of this study was to examine the impact of maternal circulating inflammatory mediators ([TNF]-α, [IL]-6, and Leptin) on placental fatty acid metabolism in GDM pregnancies.

**Methods:**

Fasting maternal blood and placental tissues were collected at term deliveries from 37 pregnant women (17 control and 20 GDM). Molecular approach techniques as radiolabeled lipid tracers, ELISAs, immunohistochemistry and multianalyte immunoassay quantitative analysis, were used to quantify serum inflammatory factors’ levels, to measure lipid metabolic parameters in placental villous samples (mitochondrial fatty acid oxidation [FAO] rate and lipid content [Triglycerides]), and to analyze their possible relationships. The effect of potential candidate cytokines on fatty acid metabolism in *ex vivo* placental explants culture following C-section a term was also examined.

**Results:**

Maternal serum IL-6, TNF-α and leptin levels were significantly increased in GDM patients compared with control pregnant women (9,9±4,5 vs. 3,00±1,7; 4,5±2,8 vs. 2,1±1,3; and 10026,7±5628,8 vs. 5360,2±2499,9 pg/ml, respectively). Placental FAO capacity was significantly diminished (~30%; p<0.01), whereas triglyceride levels were three-fold higher (p<0.01) in full-term GDM placentas. Uniquely the maternal IL-6 levels showed an inverse and positive correlation with the ability to oxidize fatty acids and triglyceride amount in placenta, respectively (r= -0,602, p=0.005; r= 0,707, p=0.001). Additionally, an inverse correlation between placental FAO and triglycerides was also found (r=-0.683; p=0.001). Interestingly, we *ex vivo* demonstrated by using placental explant cultures that a prolonged exposure with IL-6 (10 ng/mL) resulted in a decline in the fatty acid oxidation rate (~25%; p=0.001), along to acute increase (2-fold times) in triglycerides accumulation (p=0.001), and in lipid neutral and lipid droplets deposits.

**Conclusions:**

Enhanced maternal proinflammatory cytokines levels (essentially IL-6) is closely associated with an altered placental fatty acid metabolism in pregnancies with GDM, which may interfere with adequate delivery of maternal fat across the placenta to the fetus.

## 1 Introduction

Gestational diabetes mellitus (GDM) is the most prevalent metabolic disorder during gestation, and is defined as a carbohydrate intolerance of variable severity with onset or first recognition in pregnancy, independently of the treatment used for their control postpartum and evolution (1). In normal pregnancy, a physiological state of insulin resistance (IR) is required to preferentially direct maternal nutrients toward the fetoplacental axis, allowing appropriate growth of the fetus. When women develop GDM, IR is more pronounced and can disrupt the intrauterine milieu. This altered intrauterine environment has long been associated to metabolic, vascular, and/or inflammatory dysregulation, which are generally characterized by a mild hyperglycemia, dyslipidemia and augmented inflammation, especially in the third trimester of gestation (2).

In fact, GDM is characterized by an acute inflammatory response mediated by circulating cytokines, which in turn are involved in the development of insulin resistance (3, 4). Given its natural intermediate situation, the placenta becomes a regulatory target organ for these maternal environmental disorders, been exposed itself to the influence of potential harmful factors, such as hormones, cytokines, growth factors, and certain substrates present in the maternal circulation. This proinflammatory status may directly influence placental development and function from the onset of GDM (5, 6), and further may indirectly influence fetal metabolic programming for later disease (7, 8). Furthermore, the placenta is able to synthesize and release certain inflammatory mediators (9), thus contributing to low-grade systemic inflammation in uncomplicated pregnancies (10, 11).

In addition to a pronounced elevation of lipids in the maternal circulation, previous studies have mostly described the presence of an altered pro-inflammatory state characterized by increased levels of certain cytokines as IL-6 and TNF-α, and adipokines such as adiponectin and leptin, in maternal blood from pregnant women who develop GDM (12, 13). These inflammatory mediators are associated with the transport of nutrients and, hence, lipid metabolism regulation in other tissues (14–16). Interestingly, maternal cytokines have been correlated with fetal adiposity, independently of maternal pre-pregnancy BMI (17). These data suggest the possibility that pro-inflammatory cytokines can affect fatty acid flux across the placenta, compromising adequate fetal development and growth.

On the other hand, the placenta *per se* is metabolically active and may be severely affected under an impaired metabolic milieu. In this line, it has been revealed that the presence of a hyperglycemic condition is able to alter placental lipid pathways, including an inactivation of the ability to oxidize fatty acids and an increase in fat accumulation (18). This situation may contribute to the onset of excessive fat accumulation in the placenta, leading to a development of a lipotoxic injury and metabolic dysfunction, as also has recently been shown in maternal obesity (19), and may result in negative effects on the fetus at the perinatal and postnatal periods.

We know that GDM is accompanied by significant inflammatory dysregulation compared to normal pregnancy and we also know that high proinflammatory cytokines levels stimulate fatty acid accumulation in cultured human trophoblast cells (20). These findings have led us to the idea that placental metabolic function may be influenced by an altered cytokine profile. This augmented maternal inflammatory environment may be closely associated to abnormal placental lipid metabolism, being a possible determinant of the increased incidence of adverse perinatal outcomes in diabetic pregnancies.

In this study, we aimed to further investigate the impact of key inflammatory mediators on the regulation of placental fatty acid metabolism from pregnancies complicated by gestational diabetes. To this end, we hypothesized that increased maternal cytokines IL-6, TNF-α and leptin are associated *in vivo* with a reduced ability to oxidize fatty acids and, consequently, with a fat accumulation in form of triglycerides in the placenta. In addition, we planned to use an *ex vivo* study model in placental explants´ cultures to test the effect of serum pathophysiological levels of cytokines on lipid metabolism.

## 2 Materials and methods

### 2.1 Study population

A prospective cohort of sixty pregnant women followed in the Obstetrics and Gynecology Unit, at “Puerta del Mar” University Hospital of Cádiz, were initially enrolled in the third trimester of pregnancy. They were divided into two groups: 34 control women and 26 women with GDM. GDM diagnosis was made according to following inclusion criteria: previous normal glucose tolerance and diagnosis of GDM in the second or third trimester of gestation, according to the criteria of the National Diabetes Data Group (two-step approach and 100 g-OGTT with two or more values above 105, 190, 165 and 145 mg/dL after 0, 60,120 and 180 min) (21) that have been accepted by the Spanish Group of Diabetes in Pregnancy (22). Afterwards, the following exclusions criteria were established in all women: age under 18, smokers, multiple gestation, preterm deliveries (<37 weeks gestation), history of hypertensive disorders of pregnancy, chronic conditions (e.g., chronic hypertension, polycystic ovarian syndrome, inflammatory bowel disease, and chronic inflammatory conditions), thyroid disorders, pregestational diabetes, or lack of informed consent. After this screening, a total of 17 women without pregnancy complications participated in the control group and 20 women in the group with GDM, all others being excluded.

Demographic and anthropometric data including weight, height, previous body mass index, gestational age at delivery, mode of delivery, placental weight, and medical history were collected. Neonatal anthropometric measurements were performed immediately after delivery, including birth sex and birth weight. Also, information concerning clinical and metabolic status was obtained.

Written informed consent was signed from all pregnant women after explanation of protocol and testing procedures (maternal blood sampling in late pregnancy and placenta collection at delivery). This study was approved by the Human Ethics Committee of "Puerta del Mar" University Hospital (Cadiz, Spain). The confidentiality and anonymity of the data was guaranteed during the study.

### 2.2 Blood sample collection and analysis

After overnight fasting, maternal venous blood and fetal umbilical cord samples of all participants were collected in serum (gelose) and plasma (EDTA) tubes, and then were centrifuged at 1500×g for 10 minutes at 4 °C. Every fraction was immediately frozen at -80°C until analysis.

Plasma samples were processed and analyzed by clinical technical specialists at the clinical laboratory. Levels of plasma glucose, triglycerides, total cholesterol, low-density lipoprotein cholesterol (LDL-c) and high-density lipoprotein cholesterol (HDL-c) were analyzed in the modular DPD autoanalyzer (Roche Diagnostics, Manheim, Germany). Hemoglobin A_1c_ was quantified in the Cobas Integra 7000 autoanalyzer (Roche Diagnostics, Manheim, Germany). Insulin was measured in a Roche Analytics E170 analyzer (Roche Diagnostics, Manheim, Germany).

In the collected serum samples, we evaluated the levels of IL-6, TNF-α and leptin were quantified in our research laboratory (Investigation Unit, PMUH, Cádiz) using commercial kits for multiplex analysis (Procarta®114 Immunoassays Human multiplex, Affymetrix, Santa Clara, CA, USA) by Luminex equipment based on X-map technology and following the manufacturer’s instructions. The sensitivities of the tests were 0.05 pg/mL for TNF-α, 0.12 pg/mL for IL-6, 0.085 ng/mL for leptin. The intraassay coefficient of variation were 4,85 % for TNF-α, 7,853 % for IL-6, and 8,245% for leptin. Moreover, an ASC-ACOD assay (Biosystems, Barcelona, Spain) was employed for the quantitative determination of free fatty acids (FFA) using enzymatic colorimetry.

### 2.3 Placental sample collection and preparation

After a term delivery, all placentas included in the study were quickly placed on ice, transported to the laboratory within 15 min of delivery, and weighed with the umbilical cord. Then, decidual tissue and large vessels were removed from placenta by blunt dissection on aseptic culture conditions. Subsequently, small fragments of placental villous were separated from the central area of the placenta, near to the umbilical cord, rinsed in cold PBS to remove attached blood. Placental villous samples were either stored at −80 °C until further final processing and analysis or used in fresh for *ex vivo* culture assay.

### 2.4 Materials and reagents

Cell culture reagents, as RPMI-1640 medium without glucose, fetal bovine serum (FBS) and penicillin/streptomycin mix, were purchased from Invitrogen/Gibco (California, USA). The [9,10-^3^H]-palmitic acid, [^3^H]-H_2_O and liquid scintillation counting solution were from PerkinElmer, (Massachusetts, USA). Essentially fatty acid-free bovine serum albumin (BSA), palmitic acid and HPLC-grade acetone was from Sigma-Aldrich (Madrid, Spain). Recombinant human IL-6 was acquired from Peprotech (London, UK).

### 2.5 FA oxidation assay

FAO analysis was performed in placental villous samples as described previously (23) and corrected for the sample weight per assay. Briefly, freshly isolated placenta fragments (~100 mg) were incubated in RPMI-1640 culture medium supplemented with 5 mmol/l glucose, 10% fetal bovine serum (v/v) and 1% penicillin/streptomycin mix in the presence of 1.25% fatty acid-free BSA (v/v), 0.1 mmol/L unlabeled palmitate, and 18,500 Bq/mL [9,10 ^3^H]-palmitate (100 mM) for 18 hours at 37°C under a humidified atmosphere of 5% CO_2_/95% O_2_. Tritiated water ([^3^H]_2_O), as oxidized palmitate marker, was determined in the collected medium by vapor-phase equilibration method of Hughes et al. (24), after transferring it to a scintillation vial and further analyzing it by a scintillation count. All samples were tested in triplicate, and the FAO data were expressed as nmol palmitate/mg tissue/hour.

### 2.6 Placental triglycerides quantification assay

The determination of triglyceride content in human placenta was performed as previously described by Visiedo et al (25), and normalized for the total proteins per assay. Frozen placental villous (~20 mg), from both control and GDM pregnant women, were homogenized in 400 μl HPLC-grade acetone using Dounce tissue homogenizer. Afterwards, small volumes of acetone-extracted lipid suspension were used, after an overnight shaking incubation, to quantify triglyceride levels by a Triglyceride Reagent Kit (Biosystems, Barcelona, Spain). The protein content was measured in homogenized sample according to BCA Kit (Thermo Scientific, Madrid, Spain). All samples were tested in duplicate, and the TG results were expressed as mg of TG/mg of tissue protein. The intraassay coefficient of variation was 8,4 %.

### 2.7 *Ex vivo* fatty acid metabolism study in placental explants culture

Four *p*lacentas were used to evaluate the effects of pathophysiological concentrations of proinflammatory cytokine IL-6 in placental explants culture from normal pregnancies following C-section at term with no labor. Isolated villous explants in fresh were incubated in culture media in the same conditions described in the absence (lean) or presence of IL-6 (+10 ng/mL). The effects of *ex vivo* IL-6 exposure on placental FAO rates and TGs content were determined as described above. Moreover, neutral lipids and lipids droplets were histologically visualized in fresh villous tissue that were previously fixed in 10% formalin and frozen. Then, blocks were cut by a cold cryostat and placenta cryosections (10 µm) were obtained and stained by Oil Red O (ORO) stain kit (Abcam, Cambridge, UK), following the manufacturer’s instructions and using Mayer′s hematoxylin solution as counterstain for cell nuclei and tissue morphology. Slides were prepared for the capture of bright-field images using a light microscope (Nikon Eclipse 90i) at x40 magnification.

### 2.8 Statistical analysis

Statistical analysis of data was performed using the SPSS software (SPSS, Chicago, IL, USA). Distributions were checked with a histogram and the Kolmogorov-Smirnov test. All data in tables were presented as mean ± Standard Deviation (S.D.) and in figures as mean ± standard error of the mean (S.E.M.). Mann Whitney’s U test or Student’s t-tests were used to compare study variables between GDM patients and control women. Differences were considered statistically significant at P value <0.05. Linear and logistic regression analysis was used to evaluate the degree of relationship among the variables of interest (Pearson correlation coefficient). Statistically, a moderate relationship was assigned with a p value<0.05 and strong relationship with p value<0,01.

### 2.9 Study limitations

This study has some aspects that should be addressed. The general limitations of the study were those derived from its design as it is a cohort study carried out in a single center, without randomization and without being blind to any of the parts. Other limitation was that we did not distinguish between insulin-treated and diet-treated GDM women, so exogenous drug administration could influence inflammatory effects, although in our cohort only 35% of GDM women needed insulin therapy.

## 3 Results

### 3.1 Anthropometric, demographic, and metabolic characteristics in the study population

Demographic and baseline data, as well as perinatal parameters of all women who took part in the study are listed in **Table 1**. It is important to note that there were statistically significant differences between two groups only in terms of pre-pregnancy BMI and placental weight, being significantly higher in GDM group (p<0.05). In contrast, no differences were found in respect of newborn’s weight. In relation to metabolic and clinical characteristics, there were statistically significant differences in terms of maternal TGs and FFA, being significantly higher in GDM group (p<0.05). At this time, no significant differences were found in other lipids variables (e.g., total cholesterol, LDL-cholesterol, and HDL-cholesterol) or the levels of glycosylated hemoglobin between the two groups. Lastly, there were significant differences in maternal glucose levels and a similar trend was found for maternal insulin (**Table 2**).

**Table 1:** Anthropometric, demographic, and perinatal characteristics of the assessed pregnant women.

	Control (n=17)	GDM (n=20)	p value
Delivery mode (vg/cs)	*13/4*	*13/7*	
Maternal age (yrs)	*30,8±3,3*	*33,7±5,3*	*0,07*
Gestational age at delivery (wks)	*39,1±1,4*	*38,6±1,6*	*0,20*
Pre-pregnancy BMI (kg/m²)	*23,1± 3,1*	*26,9±5,3*	0,02*
Placental weight (gr)	*505±83*	*620±78*	0,006**
Newborn Birthweight (gr)	*3264±322*	*3258±507*	*0,53*
Birth sex (male/female)	*7/10*	*10/10*	

GDM, gestational diabetes mellitus; BMI, body mass index; vg, vaginal delivery; cs, Cesarean section. In normal variables, the data are listed as mean (± standard deviation). In qualitative variables, the sample size (n) is shown. *p value< 0.05 vs. control group; **p value< 0.01 vs. control group.

**Table 2:** Maternal clinical and metabolic parameters of the studied pregnant women.

	Control (n=17)	GDM (n=20)	p value
Maternal glucose (mg/dL)	*73,0±10,1*	*79,8±9,5*	*0,07*
Maternal triglycerides (mg/dL)	*201,7±67,2*	*274,2±84,0*	0,01*
Maternal total cholesterol (mg/dL)	*257,7±45,6*	*250,2±51,6*	*0,68*
Maternal HDL cholesterol (mg/dL)	*67,7±20,3*	*62,7±14,6*	*0,45*
Maternal LDL cholesterol (mg/dL)	*147,9±36,5*	*144,3±43,7*	*0,81*
Maternal total cholesterol/HDL cholesterol ratio	*4,0±1,1*	*4,1±1,0*	*0,90*
HbA1C (%)	*5,2±0,2*	*5,3±0,3*	*0,16*
Maternal insulin (pmol/L)	*7,9±3.7*	*11,8±5,3*	*0,07*
Maternal free fatty acid (mEq/L)	*0,37±0,1*	*0,76±0,3*	0,001**

GDM, Gestational diabetes mellitus; BMI, Body mass index; HbA1C, hemoglobin A1c. HDL, High-density lipoprotein; LDL, Low-density lipoprotein. Data are listed as mean (± standard deviation). *p value< 0.05 vs. control group; **p value< 0.01 vs. control group.

### 3.2 Inflammatory markers in the study population

There were significant differences between control and GDM women in maternal serum proinflammatory cytokines levels: IL-6 (3,0±1,7 vs 9,97±4,5 pg/mL; p=0.001) and TNF-α (2,1±1,3 vs 4,5±2,8 pg/mL; p=0.002), and adipokine levels: leptin (5360,2±2499,9 vs 10026,7±5628,8 pg/mL; p=0.002). Meanwhile, no differences were found for these cytokines in cord blood serum between both groups (**Table 3**).

**Table 3:** Inflammatory cytokines levels in control and GDM groups.

	Control (n=17)	GDM (n=20)	p value
Maternal IL-6 (pg/mL)	*3,0±1,7*	*9,9±4,5*	0,001**
Maternal TNF-α (pg/mL)	*2,1±1,3*	*4,5±2,8*	0,002**
Maternal Leptin (pg/mL)	*5360,2±2499,9*	*10026,7±56285,8*	0,002**
Cord IL-6 (pg/mL)	*4,4±3,8*	*7,9±5,5*	*0,09*
Cord TNF-α (pg/mL)	*1,5±0,7*	*1,4±0,7*	*0,67*
Cord Leptin (pg/mL)	*3421,2±1204,2*	*4192,1±1995,5*	*0,30*

GDM, gestational diabetes mellitus; IL-6, interleukin 6; TNF-α, tumor necrosis factor alpha. Data are listed as mean (± standard deviation). **p value<0.01 vs. control group.

### 3.3 Placental fatty acid metabolism parameters

**Figure 1:**
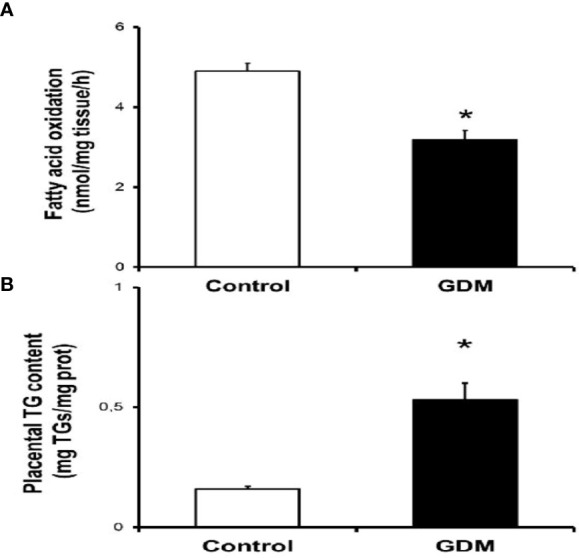
Altered lipid metabolism in placentas from pregnant GDM. **(A)** Placental fatty acid oxidation assay. Placentas from women with no pregnancy complication (control, n=10) and gestational diabetic women (GDM, n-10) were employed to obtain villous samples as described in "Methods" section. Values are Mean ± S.E.M. for 10 independent experiments in triplicate. Significance is defined as *p<0.05 relative to control group. **(B)** Placental triglyceride content assay. Frozen placental tissues (-20 mg) from control (n=10) and GDM group (n=10) were used to quantify triglyceride levels as described in the "Methods" section. Values are Mean ± S.E.M. for 10 independent experiments in triplicate. *p<0.05 relative to control group.

Of all the pregnant women recruited for this study (n=37), placentas could only be collected from 20 women (10 controls and 10 GDM cases) at the time of delivery to perform the FA metabolism measurements. Here, we measured the FAO capacity in placental explants from control and GDM women enrolled in the study. As shown in **Figure 1A**, oxidation of [^3^H]-palmitate was 30 % higher in placentas of women with GDM compared with control group (4.89±0.6 vs 3.19±0.73 nmol/mg tissue/h; p<0.01). At this time, extremely higher placental triglyceride concentrations (0.15±0.03 vs 0.53±0.21 mg TGs/mg tissue; p<0.01) were found in pregnant women with GDM in comparison with control group at the time of delivery (**Figure 1B**).

### 3.4 Relationship analysis between lipid metabolism and cytokines variables

**Figure 2:**
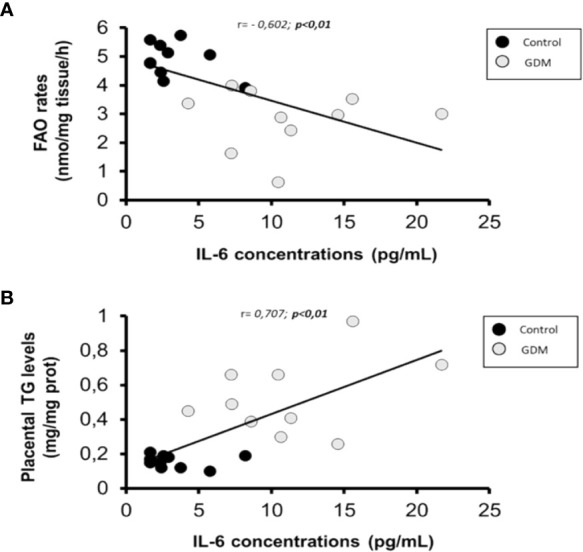
Scatterplot of maternal serum IL-6 levels in comparison with placental FAO rates and TGs content. Pearson correlation coeficient are shown, where a negative value indicates an inverse relationship **(A)** and a positive value a direct relationship **(B)**. Statistical significance was determined when p<0,01. n=20 (10 GDM and 10 control women).

**Figure 3:**
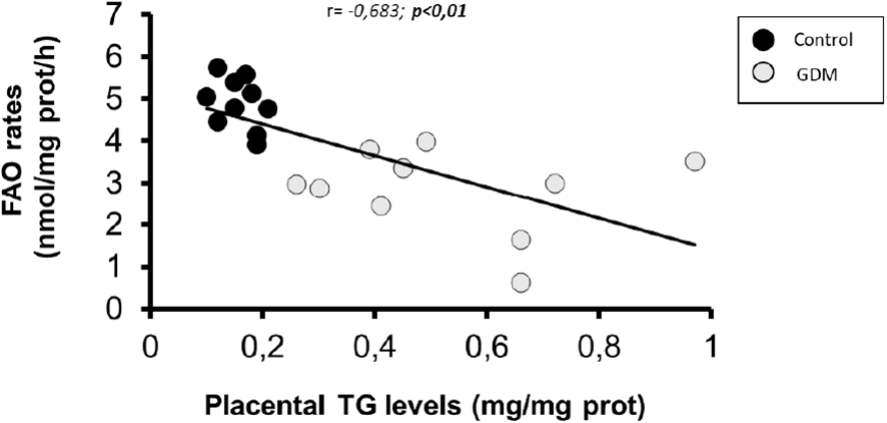
Scatterplot of placental FAO rates in comparisgn with IGs content. Pearson correlation coeficient is shown, where a negative value indicates an inverse relationship. Statistical significance was determined when p<0,01. n-20 (10 GDM and 10 control women).

As shown in **Table 4**, only significant correlations were found between maternal serum IL-6 and lipid metabolism parameters in villous samples. As is shown in **Figures 2A, B**, there was a significant indirect correlation between IL-6 and FAO rates (r=-0.602; p=0.005), and direct between IL-6 and TGs content (r= 0,707; p=0.001) correlation, respectively. Interestingly, we also observed a significant inverse correlation between placental FAO and TGs (r= -0.683; p=0.001), as shown in **Figure 3**.

**Table 4:** Relationships between placental lipid metabolism parameters and maternal inflammatory mediator’s levels.

	Placental FAO rate
**IL-6 (pg/mL)**	r= -0,602; p=0,005**
**TNF-a (pg/mL)**	*r= -0,426; p=0,061*
**Leptin (pg/dL)**	*r= -0,403; p=0,078*

	**Placental TGs accumulation**
**IL-6 (pg/mL)**	r= 0,707; p=0,001**
**TNF-a (pg/mL)**	*r= 0,383; p=0,096*
**Leptin (pg/dL)**	*r=-0,343; p=0,138*

	**Placental FAO rate**
**Placental TGs**	R= -0,683; p=0,001**

GDM, gestational diabetes mellitus; IL-6, interleukin 6; TNF-α, tumor necrosis factor alpha; FAO, fatty acid oxidation; TG, triglycerides. The correlation (r) is significant at the 0.01 level (bilateral). **p<0,01.

### 3.5 *Ex vivo* effects of pathophysiological IL-6 levels on placental fatty acid metabolism

**Figure 4:**
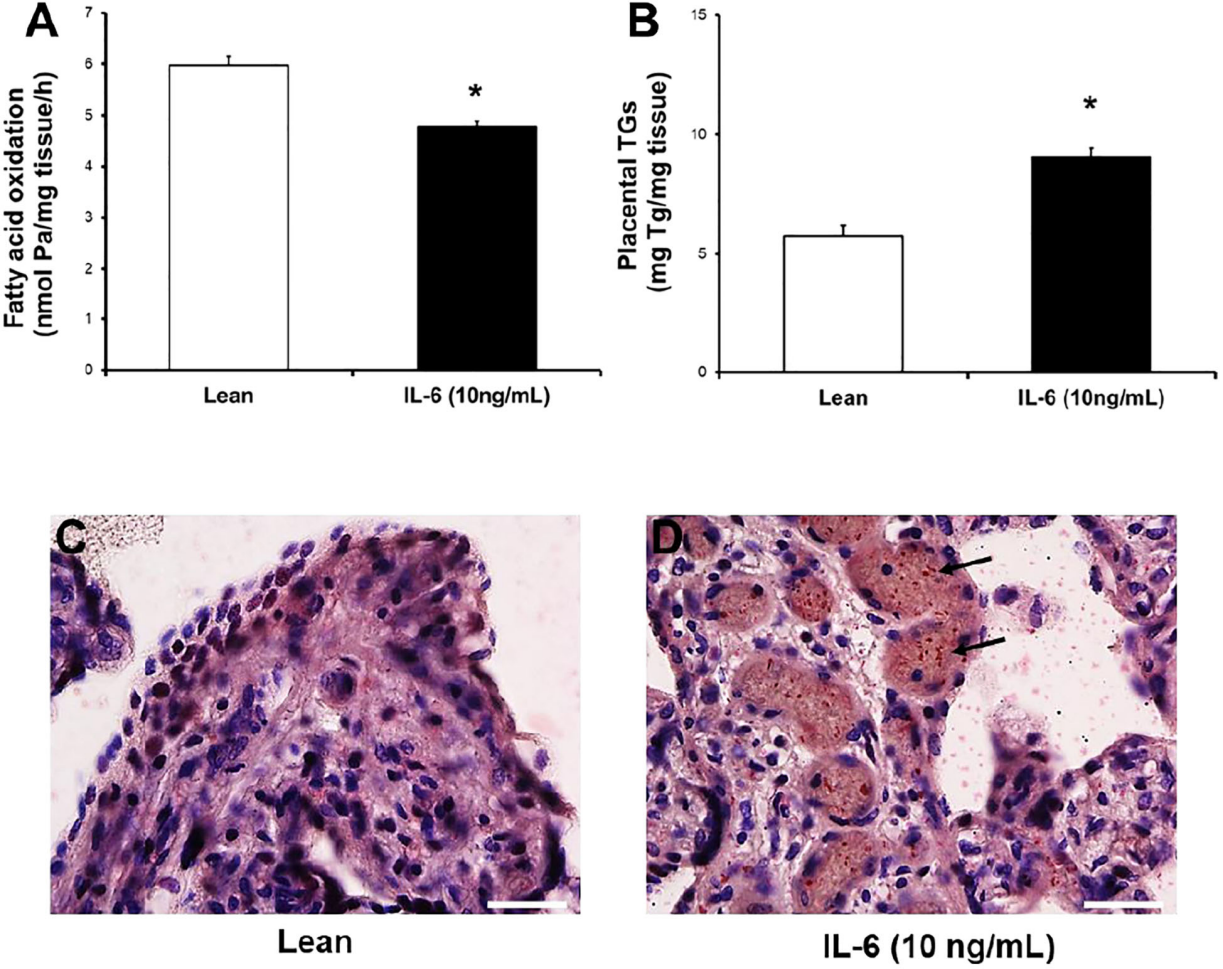
IL-6 significantly reduces [^3^H]-palmitate oxidation capacity and enhances lipid content in human placental explants. **(A, B)** Effect of IL-6 on FAO capacity and TGs accumulation. Placental villous from control women (n=4; **Table 5**) were incubated in the absence (lean) or presence of IL-6 (+10 ng/mL) for 18 h. Afterward, [3H]-water and TGs levels was determined by methods described. Values are mean ± S.E.M. for independent experiments in triplicate and duplicate, respectively. *p<0.05 compared with lean by Mann-Whitney U test. **(C, D)** Fresh frozen placental sections from untreated (lean) and treated (+IL-6) were fixed and stain lipids with Oil Red "O", as described in Methods section. Representative microphotography from two independent experiments is shown (Scale bars, 25 µm; 40x magnification, Nikon eclipse 90i). Blue stain (nuclei and tissue 3 morphology) and red stain (neutral lipids or lipid droplets).

As a result of the determinations of serum levels of inflammatory mediators between the two groups, and given that only IL-6 was found to have a significant direct (FAO) and indirect (TGs) relationship with the two markers of lipid metabolism, we next considered conducting an *ex vivo* experimental study with fresh explants culture to test the effect of elevated levels of recombinant IL-6 found in serum of pregnant women with GDM on fatty acid metabolism, using a representative subset of placentas (n=4) from control women (**Table 5**). Therefore, we measured the effect of chronic exposure (18 h) to pathophysiological levels of IL-6 (10 ng/mL) on FAO and TG content in placental explants. As shown in **Figures 4A, B**, IL-6 significantly reduced the FAO rate by ~25% in placental fatty acid oxidation (5,97± 0,51 vs 4,77±0,3 nmol/mg protein/h; p=0.001) and increased considerably triglycerides levels (5,75±0,92 vs 9,08±0,69 mg TGs/mg protein; p=0.001). Moreover, neutral lipids and lipid droplets deposits (**Figure 4C**, **4 D**) were found largely in villous cryosections exposed with 10 ng/mL IL-6 compared to untreated, following ORO staining.

**Table 5:** Demographic and gestational characteristics of included pregnant women in *ex vivo* placental assays.

	Control (n=4)
Delivery mode	*C-section*
Maternal age (yrs)	*33,2±1,5*
Gestational age at delivery (wks)	*38,7±0,8*
Pre-pregnancy BMI (kg/m²)	*23,9±0,6*
Placental weight (gr)	*578,5±73,4*
Newborn Birthweight (gr)	*3115,0±257,7*

Values are listed as means ± (standard deviation). BMI, body mass index.

## 4 Discussion

In this study, we showed by analysis in blood that women with GDM had elevated serum levels of the proinflammatory mediators (IL-6, TNF-α) and adipokines (leptin) in the third trimester of pregnancy. No differences were detected in the levels of these cytokines in the blood collected from the cord. As a result of this, a statistically significant negative and positive correlation between increased IL-6 levels and decreased FAO capacity, and the accumulation of TGs in placental tissue was found, respectively. In order to demonstrate the direct role of IL-6 in these placental metabolic alterations, placental villous explants from healthy pregnant women were exposed to recombinantproinflammatory cytokines (only IL-6 and TNF-α), resulting in a similar effect to that observed in vivo, characterized by the presence of reduction in fatty acid oxidation rate and an augmentation in the storage of placental neutral lipids and TGs.

Abnormal maternal metabolic and inflammatory profiles have long been associated with pregnancy disorders such as maternal diabetes or obesity (6, 26). Thus, elevated maternal concentrations of nutrients and molecules as glucose, free fatty acids (FFA), insulin or cytokines may affect placental metabolic function, including alterations in lipid storage, metabolism, and transport. This impaired *in utero* scenario may underlie changes in fetal development and growth, and in offspring long-term metabolic health. In this line, the regulation of placental lipid metabolism has long been recognized as a critical mechanism at the maternal-fetal interface during pregnancy, particularly from the third trimester of gestation (27).

Our previous results showed that high glucose levels modify the metabolic distribution of fatty acids in human placenta shifting flux from oxidation to the esterification pathway and accumulation of placental triglycerides (28). Nevertheless, the influence of damaged maternal inflammatory profile on placental metabolic function has not yet been elucidated. The present study provides some novel information about it. Thus, we first showed that an altered placental lipid metabolism is associated with enhanced maternal cytokines levels (essentially IL-6) present in serum of GDM women in late gestation, including a diminished placental fatty acid β-oxidation capacity and an excess FFA accumulation in the form of TGs, which may limit the amount of maternal lipid transferred to the fetus across of the placenta in diabetic pregnancies.

Overall, obese and GDM mothers present both an enhanced maternal lipid profile and inflammatory response (12, 29–32), even remaining so throughout of pregnancy (33), although some recent reviews have suggested that exist a certain inconsistency about the exact values of circulating proinflammatory cytokines in GDM (34). In our cohort study, the results in maternal lipid profile, such as increased triglyceride and free fatty acid levels, and unchanged total cholesterol, HDL-cholesterol, and LDL-cholesterol levels in women with GDM compared with those without GDM, were in consonance with works of the previous meta-analysis analysis (30, 33). Additionally, these results are along the same line with other studies, showing also a greater proinflammatory pattern in women with GDM compared to control women, including elevated IL-6, TNF-α and leptin levels (12).

It has been demonstrated that certain proinflammatory cytokines, as IL-6 and TNF-α, and adipokines, as leptin, contribute significantly to the development of abnormal lipid metabolism, in particular FA metabolism (35). Some exert a positive metabolism action, while others make a negative role which is linked to the occurrence of metabolic dysfunction. Thus, both IL-6 and TNF-α have been found as key modulators in fat metabolism in skeletal muscle (14, 15). On the other hand, leptin, an adipocyte-derived hormone, promotes FA oxidation, glucose uptake and prevents the accumulation of lipids in non-adipose tissues (36, 37). In general, when FFAs enter in cells either can be converted to TGs for storage or to undergo β-oxidation to be used as energy source at mitochondrial level. However, when an excess of saturated FFAs occurs, the cell becomes overloaded and full conversion to TGs or complete β-oxidation is impossible, resulting in the formation of toxic lipids (so called lipotoxicity) (38). In our study, analysis of placental lipid metabolism confirmed the presence of this phenomenon through a reduced placental FAO, accompanied by an excessive fat accumulation in form of triglycerides in placentas from full-term GDM pregnancies compared to non-GDM women.

The placenta is also target of cytokines and displays a marked metabolic activity, which is severely affected by the intrauterine milieu of diabetic and/or obese women. A previous study of our group showed that pro-inflammatory cytokine hepatocyte growth factor (HGF) is elevated in amniotic fluid of obese women and closely linked to the regulation of glucose and lipid placental metabolism, through excessive triglyceride accumulation mediated by inhibition of fatty acid oxidation and increased fatty acid esterification (25). These findings appear to be in accordance with our results between maternal lipid metabolism and circulating maternal cytokines, and with the presence of altered NO-mediated oxidative status in GDM women (39). Herein, we first found that only maternal serum IL-6, but no TNF-α or leptin negatively correlate with placental FAO capacity and TGs content at term. These findings highlight the potential metabolic role of IL-6 as a potential modulator of lipid metabolism associated with pregnancies complicated with GDM and with the development of placental metabolic dysfunction, since free fatty acids can accumulate in the body organs including the placenta if their levels remain continuously elevated, ultimately resulting in lipotoxic damage. In turn, there was an indirect correlation between FAO and TGs. These data therefore provide a mechanistic link between FAO incapacity and fat accumulation in form of triglycerides in placentas from GDM pregnancies.

To test whether pathophysiological levels of IL-6 affect lipid metabolism in human placenta, we examine by a *ex vivo* model that uniquely a prolonged exposure of IL-6 (10 ng/mL) directly disrupted the oxidation rate of tritiated palmitate in explants culture and increased fat deposits. No differences related to FA metabolism were observed using pathophysiological TNF-α (5 ng/mL) levels found in circulating maternal GDM (data not shown). These results partly resemble other similar studies examining the role of cytokines IL-6 and TNF-α in the regulation of fat metabolism, where the pathophysiological effects of cytokines were evaluated *in vitro* primary cell or in *ex vivo* models with tissue biopsies. Thus, Lager et al. showed that elevated IL-6, but no TNF-α, stimulated fatty acid accumulation trophoblast primary culture, although without a corresponding increase in the expression of proteins or mRNAs related to fatty acid transport (20); on the other hand, Bruce et al. demonstrated that IL-6 increased FAO and attenuated insulin´s lipogenic impact, whereas TNF-α exerted no effect on FAO but increased FA incorporation into diacylglycerides (DAG) in rat soleus model (40). Taken together, these results seem to indicate the presence of tissue-specific differences in the mechanistic action of cytokine-induced lipid metabolism.

## 5 Conclusion

We first found that an altered placental fatty acid metabolism is associated with enhanced maternal cytokines levels (essentially IL‐6) present in serum of GDM women in late gestation, including a diminished placental fatty acid β‐oxidation capacity and an excess FFA accumulation in form of TGs. This finding highlights that an exacerbated proinflammatory environment in pregnancy would be closely related to the emergence of inadequate transport of essential lipids to the fetus and, consequently, to the onset of placental lipotoxic damage. Likewise, this scenario would lead to the occurrence of late adverse outcomes mediated by metabolic programming phenomena and associated with a metabolically stressful maternal environment, as it occurs in obesity or maternal diabetes. Therefore, these new results open a new window of knowledge about a still unknown relationship between inflammation and placental lipid metabolism, and that needs to be further comprehensively evaluated.

## Author’s note

Partial results of this study were presented at the Spanish Society of Biochemistry and Molecular Biology congress (SEBBM) celebrated in Madrid, Spain (Ref. P-07m1-R07-05; 6th September, 2013).

## Data availability statement

The raw data supporting the conclusions of this article will be made available by the authors, without undue reservation.

## Ethics statement

The studies involving human participants were reviewed and approved by CEI/11, October 2011. The patients/participants provided their written informed consent to participate in this study.

## Author contributions

Conceptualization: FB, RM, and FV. Formal analysis: FV. Funding acquisition: FB. Investigation: FV, LV-F, JB-M, JA, RQ-P. Methodology: RM, FB, and FV. Project administration: FB. Visualization: FV. Writing—original draft: FV, RM, and FB. Writing-review and editing: all authors. All authors have read and accepted the final version of the manuscript. All authors contributed to the article.
